# Characterization of the biochemical and behavioral effects of cannabidiol: implications for migraine

**DOI:** 10.1186/s10194-023-01589-y

**Published:** 2023-05-03

**Authors:** Rosaria Greco, Miriam Francavilla, Chiara Demartini, Anna Maria Zanaboni, Mikael H. Sodergren, Sara Facchetti, Barbara Pacchetti, Michela Palmisani, Valentina Franco, Cristina Tassorelli

**Affiliations:** 1 Unit of Translational Neurovascular Research, IRCCS Mondino Foundation, 27100 Pavia, Italy; 2 Department of Brain and Behavioral Sciences, University of Pavia, 27100 Pavia, Italy; 3 Curaleaf International, Guernsey, UK; 4 Medical Cannabis Research Group, Imperial College London, London, UK; 5 Clinical and Experimental Pharmacology Unit, Department of Internal Medicine and Therapeutics, University of Pavia, 27100 Pavia, Italy

**Keywords:** Migraine, CBD, Trigeminal hyperalgesia, Inflammation

## Abstract

Cannabidiol (CBD) is the main pharmacologically active phytocannabinoid. CBD exerts an analgesic effect in several pain models, does not have side effects and has low toxicity. The data about CBD mechanisms of action in pain and its therapeutic potential in this area are limited. Here, we tested CBD effects in animal models specific for migraine. We assayed CBD distribution in plasma and in cranial areas related to migraine pain in male Sprague Dawley rats treated chronically (5 days). Successively, we tested CBD activity on the behavioral and biochemical effects induced in the acute and the chronic migraine animal models by nitroglycerin (NTG) administration. In the acute migraine model, rats received CBD (15 mg or 30 mg/kg, i.p) 3 h after NTG (10 mg/kg i.p.) or vehicle injection. In the chronic migraine model, rats were treated with CBD and NTG every other day over nine days with the following doses: CBD 30 mg/kg i.p., NTG 10 mg/kg i.p. We evaluated behavioral parameters with the open field and the orofacial formalin tests. We explored the fatty acid amide hydrolase gene expression, cytokines mRNA and protein levels in selected brain areas and CGRP serum level. CBD levels in the meninges, trigeminal ganglia, cervical spinal cord, medulla pons, and plasma were higher 1 h after the last treatment than after 24 h, suggesting that CBD penetrates but does not accumulate in these tissues. In the acute model, CBD significantly reduced NTG-induced trigeminal hyperalgesia and CGRP and cytokine mRNA levels in peripheral and central sites. In the chronic model, CBD caused a significant decrease in NTG-induced IL-6 protein levels in the medulla–pons, and trigeminal ganglion. It also reduced CGRP serum levels. By contrast, CBD did not modulate TNF-alpha protein levels and fatty acid amide hydrolase (FAAH) gene expression in any of investigated areas. In both experimental conditions, there was no modulation of anxiety, motor/exploratory behavior, or grooming. These findings show that CBD reaches brain areas involved in migraine pain after systemic administration. They also show for the first time that CBD modulates migraine-related nociceptive transmission, likely via a complex signaling mechanism involving different pathways.

## Background

Migraine is one of the most disabling painful conditions and a common disorder [1]. Activation of the trigeminovascular system with subsequent release of calcitonin gene-related peptide (CGRP) and other pro-inflammatory mediators in the dura mater plays and key role in migraine attacks [2]. Frequent recurrence of migraine attacks lowers the nociceptive thresholds leading to an abnormal release of nociceptive molecules [3, 4]. As a result, trigeminal and thalamic neurons may become sensitized, with limited recovery between episodes, leading to chronic migraine [5, 6].

The endocannabinoid (eCB) signaling system regulates a broad spectrum of physiologic processes and it has attracted considerable attention as a potential pharmaceutical target for modulating pain perception, emotional state, reward behaviors, learning, and memory [7–9]. The modulation of the endocannabinoid system (ES) is effective in different disorders, including migraine [10].

Peripheral and global inhibitors of fatty acid amide hydrolase (FAAH) – the enzyme that degrades anandamide (AEA), one of the best-known endocannabinoids—modulate the functional status of central structures and reduce gene expression of pain mediators in migraine-specific animal models [11–13]. ES may regulate the release of several mediators, including CGRP and pro-inflammatory cytokines, by CB1 receptor activation [14] through changes in central and peripheral areas in these animal models [13, 15].

Phyto-cannabinoids, as well as synthetic cannabinoids, reduce pain, inflammation, anxiety, and depression in different animal models of diseases [16]. Cannabinoids modify functions and activity of signaling pathways that have a role in pain control. Numerous studies also suggest that exogenous cannabinoids may interact with the ES and may be relevant for migraine via multiple mechanisms [17, 18].

Although cannabinoids have been suggested as a potential migraine treatment, the evidence about their efficacy and tolerability is lacking [19].

Interestingly, several studies have demonstrated that cannabidiol (CBD) has significantly fewer side effects than its psychoactive equivalent, delta (9)-tetrahydrocannabinol (THC) [20, 21] since it is a negative allosteric modulator of CB1 receptors and a partial agonist of CB2 receptors [22, 23]. CBD has been proposed as a possible treatment for inflammatory disorders and neuropathic pain [24]. Isolated reports suggested that migraine sufferers may experience some relief with *Cannabis* [18].

CBD has demonstrated analgesic and anti-hyperalgesic effects in animal models of pain not specific for migraine, as well as anti-allodynic effects [25]. These effects may be related to an increased eCB signaling through an inhibitory action on the mechanisms of eCB degradation (i.e., the transporter and the AEA-degrading fatty acid amide hydrolase (FAAH) enzyme) [26, 27]. However, other pharmacological targets have been proposed for pain-related CBD activity. Like other cannabinoids, CBD suppresses the activity of mediators (cytokines, chemokines) and cells (macrophages and related cells) that are involved in neurogenic inflammation and therefore, in the mediation of migraine pain [28].

Taken together, these pieces of information suggest that CBD bears a potential to play a role in some of the mechanisms involved in migraine pathophysiology. However, preclinical studies that focus specifically on the efficacy of CBD on migraine are currently lacking [18].

In this study, we investigated the performance of CBD in the well-known animal models of acute and chronic migraine based on systemic nitroglycerin (NTG) administration to test whether: i) CBD administration may actually have a therapeutic potential in migraine and ii) identify the molecular mediators of this activity.

To this end, we evaluated the effect of CBD on: a) the NTG-induced trigeminal hyperalgesia at the orofacial formalin test; b) the NTG-induced anxiety-like and spontaneous locomotor activity behavior; c) CGRP plasma levels and gene expression in the brain, cytokine protein and mRNA expression, and FAAH gene expression in brain areas of interest. Finally, we also evaluated the distribution of CBD in cranial areas involved in migraine pain.

## Methods

### Animals

We used adult male Sprague–Dawley rats weighing 150–175 g. The use and handling of the animals were in accordance with the guidelines provided by the International Association for the Study of Pain [29]. The Italian Ministry of Health approved the experimental protocols (N. 691/2020-PR) and the tests were carried out in accordance with the European Convention for the Care and Use of Laboratory Animals. Rats were housed in plastic boxes in groups of two with water and food available ad libitum and kept on a 12:12 h light–dark cycle at the Centralized Animal Facility of the University of Pavia. Upon arrival, animals were habituated to the housing conditions for one week before the experimental testing. The experiments were performed in a randomized manner by an experimenter blinded to treatments. The experimental procedures were optimized to reduce animal suffering potentially related to chronic intraperitoneal administration. Humane endpoints to evaluate animal health were based on the following: a) body weight (> 20 percent weight loss) and body condition (piloerection or abnormal posture); b) decreased food/water consumption; c) behavioral changes (social isolation).

### Drugs

Nitroglycerin (NTG) (Bioindustria L.I.M. Novi Ligure (AL), Italy) was prepared from a stock solution of 5.0 mg/1.5 mL dissolved in 27% alcohol and 73% propylene glycol. For the injections, NTG was further diluted in saline (0.9% NaCl) to reach the final concentration of alcohol 6% and propylene glycol 16%. The diluted NTG is injected intraperitoneally (i.p.) at the dose of 10 mg/kg. An equivalent volume of saline (0.9% NaCl), alcohol 6% and propylene glycol 16% was used as vehicle.

Curaleaf International, UK, offered CBD derived from Cannabis plants as a refined powder. CBD purity was greater than 98%; CBD powder was dissolved in 10% polyethylene glycol 200, 10% tween 80 and saline, protected from light, and agitated until mixed. The dissolved CBD was prepared freshly before injection and delivered at two different doses, 15 mg/kg and 30 mg/kg [30].

### Experimental design

#### CBD distribution in blood and in nervous tissue

CBD was quantified in rat brain tissue and plasma samples using a previously proven online solid phase extraction (SPE) high-performance liquid chromatography (HPLC) method coupled with tandem mass spectrometry (MS/MS) [31].

**Fig. 1:**
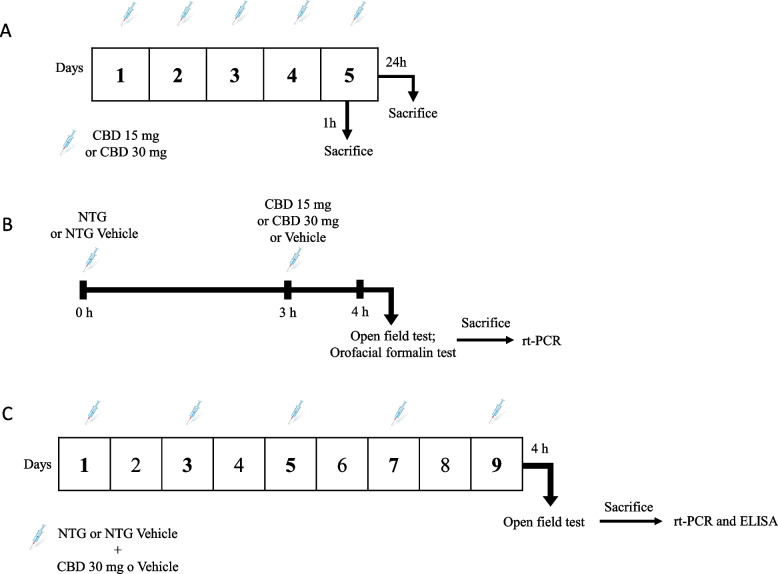
Experimental timeline for the treatment and testing procedures: **A** CBD distribution in blood and in nervous tissue; **B** Acute migraine model; **C** Chronic migraine model

Four sets of male Sprague–Dawley rats (*n* = 7 per group) received CBD 15 mg/kg or 30 mg/kg for 5 consecutive days (Fig. 1A). After treatment, the animals were sacrificed 1 h or 24 h after the last CBD administration and blood, trigeminal ganglia (TGs), meninges, cervical spinal cord (CSC), and medulla-pons were collected. Rats were sacrificed with a lethal dose of anesthetic followed by decapitation. Truncal blood was centrifuged for 15 min at 1000 g at 2—8 °C for plasma collection. CBD levels in rats’ plasma and brain samples were performed by online-SPE LC–MS/MS [31].

The samples of nervous tissue were weighed and homogenized with methanol and the internal standard cannabidiol-d3 (170 ng/mL in methanol). Then, samples were centrifuged at 17,000 g for 5 min at 4 °C, and 40 μl of supernatant was transferred into HPLC vials and injected into an SPE-HPLC–MS/MS system. Plasma samples (50 μl) were mixed with 50 µl of the internal standard cannabidiol-d3 (170 ng/ml in methanol) and 150 µl of methanol. After vortexing, the mixture was centrifuged for 10 min (16,000 g at 4 °C). The supernatant (40 μl) was injected into the HPLC–MS/MS system [31].

#### Acute migraine model

Male Sprague–Dawley rats (*n* = 7–8 per group) were used to test the effect of a single administration of CBD (doses tested: 15 mg/kg or 30 mg/kg, i.p.) in the NTG-based animal model of acute migraine. NTG was prepared as previously described [11] and administered i.p. 4 h before testing and/or ex vivo analysis at the dose of 10 mg/kg. CBD was administered 3 h after NTG (or vehicle) injection. Four hours after NTG (or vehicle) administration, rats underwent the open field test and orofacial formalin test. All rats were sacrificed at the end of the test to evaluate gene expression (cytokines, CGRP) in medulla-pons (bregma, 13.30 to 14.60 mm), CSC (C1-C2), trigeminal ganglion (TG) (Fig. 1B).

#### Chronic migraine model

Male Sprague–Dawley rats (*n* = 7 per group) were used to test the effect of chronic CBD in the animal model of chronic migraine. Since we did not see significant differences between the two doses of CBD in the acute paradigm, we used the higher CBD dose (30 mg/kg, i.p.) or its vehicle (1 ml/kg, i.p.) which was co-administered with NTG (10 mg/kg, i.p.) or its vehicle to the rats every two days over nine days [32]. On the final testing day, CBD was administered three hours after NTG (or vehicle) and the rats were evaluated at the open field test 1 h later the last administration. Rats were then euthanized to evaluate CGRP serum levels, FAAH gene expression, and cytokine protein levels in medulla-pons, CSC, and TG (Fig. 1C).

#### Open field test

Measurement of the distance travelled in the entire arena, the time spent in the center, and rearing behavior were used to assess locomotor activity, anxiety, and exploration, respectively [33]. Spontaneous grooming behavior was evaluated as an indicator of increased nociception [15]. The evaluation of these parameters was performed by a trained observer blinded to treatment condition, using the ANY-Maze software (Ugo Basile, application version 4.99 g Beta). By means of the ANY-Maze software, the open-field arena was divided into 16 square units, identifying 4 squares as the center and 12 squares along the outer perimeter as the periphery.

#### Orofacial formalin test

Rats were acclimatized to the test chamber in the days before the orofacial formalin test for 10 min. The observation box was a 30 × 30 × 30 cm glass chamber with mirrored sides. A camera, recording face rubbing time for off-line analysis, was located at a distance of 50 cm from the box to provide a clear view of each rat. On the test day, rats were given a subcutaneous injection of formalin (1.5%, 50 µl) into the right upper lip and face rubbing was measured counting the seconds each animal spent grooming the injected area with the ipsilateral forepaw or rear paw 0–3 min (Phase I) and 12–45 min (Phase II) after formalin injection.

#### rtPCR and ELISA evaluations

After the behavioral tests, rats were sacrificed with a lethal dose of anesthetic followed by decapitation. In the acute migraine model, meninges, medulla-pons (bregma, -13.30 to -14.60 mm), CSC, and TG ipsilateral to the formalin injection were quickly dissected out, rinsed in sterile 0.9% NaCl solution, placed in cryogenic tubes and immediately frozen in liquid nitrogen. The areas were subsequently kept at -80 °C until they were processed with rt-PCR for cytokines, inducible nitric oxide synthase (iNOS) and CGRP gene expression evaluation.

In the chronic migraine model, medulla-pons, CSC, and TG were removed, separated into right and left sides, cleaned in a cold and sterile 0.9% NaCl solution, placed in cryogenic tubes, and immediately frozen at -80 °C until. They were processed with rt-PCR for FAAH gene expression and with ELISA for the evaluation of cytokines and CGRP serum levels.

All procedures were performed under RNase-free conditions. After RNA extraction, the absorbance ratios (260/280 nm) ranged from 1.9 to 2.0 in all RNA samples, indicating no significant protein (including of blood origin) contamination. mRNA levels were measured by rt-PCR. Glyceraldehyde 3-phosphate dehydrogenase (GAPDH), whose expression remained constant in all experimental groups, was used for normalization. In Table 1 are reported the primers used for rt-PCR analysis. All samples were assayed in triplicate and the ΔΔCt method was used to investigate the differences in gene expression levels. Pro-inflammatory cytokines in the medulla-pons, CSC, and TG were measured using the ELISA kits (Diaclone Co, Besançon, France).

**Table 1:** Primer sequences

Gene	Forward primer	Reverse primer
GAPDH	AACCTGCCAAGTATGATGAC	GGAGTTGCTGTTGAAGTCA
TNF-alpha	CCTCACACTCAGATCATCTTCTC	CGCTTGGTGGTTTGCTAC
IL-6	TTCTCTCCGCAAGAGACTTC	GGTCTGTTGTGGGTGGTATC
iNOS	CCGGCTACACTTCTCCTCAC	CACGAAGCAGGGGACTACAT
CGRP	CAGTCTCAGCTCCAAGTCATC	TTCCAAGGTTGACCTCAAAG
FAAH	TTGGAGGGATGGCAGCTTTA	AAGAAAGGGTGGAGGAGCTC

Truncal blood was centrifugated for 15 min at 1000 g at 2–8 °C for plasma collection. The serum CGRP levels were assayed using a commercial ELISA kit (CGRP: Elabsciences, Houston, TX, USA). The samples' measured absorbance was compared to a standard curve using a microplate reader (Biotek, Santa Clara, CA, USA).

#### Statistical analysis

An a priori power analysis was conducted to determine the minimum sample size needed to obtain a statistical power of 0.80 at an alpha level of 0.05 (GPower 3.1). Based on our previous studies on FAAH inhibitors [11, 13] and considering that CBD may act via the inhibition of FAAH, we hypothesized a difference in mean total nociceptive response in the second phase of the orofacial formalin test (face rubbing time) of about 60 s between rats injected with NTG and vehicle and rats injected with NTG and CBD (NTG + CBD vehicle 170 ± 40; NTG + CBD = 110 ± 39). Thus, we estimated a sample size of 7 rats in each experimental group with an effect size of 1.52. However, due to the intergroup variability seen in the orofacial formalin test, we used a maximum of 8 rats per group.

Data were tested for normality utilizing the Kolmogorov–Smirnov test.

Depending on data distribution was applied parametric or non-parametric tests with post hoc analysis for multiple comparisons. A probability level of less than 5% was considered significant. Nocifensive responses, gene expression, and CBD levels were normally distributed and were therefore analyzed using the parametric one-way ANOVA followed by post hoc Tukey's multiple comparisons test or 2-way ANOVA followed by Sidak's multiple comparisons tests. Open field test evaluations, grooming time, and number of rearings were not normally distributed and we therefore used non-parametric Kruskal–Wallis's test, followed by Dunn's post-hoc test for the analysis. Data were expressed as mean ± SEM or as median and minimum and maximum values. Statistical analyses were performed using GraphPad Prism software (version 8).

## Results

### CBD accumulation in cranial areas

**Fig. 2:**
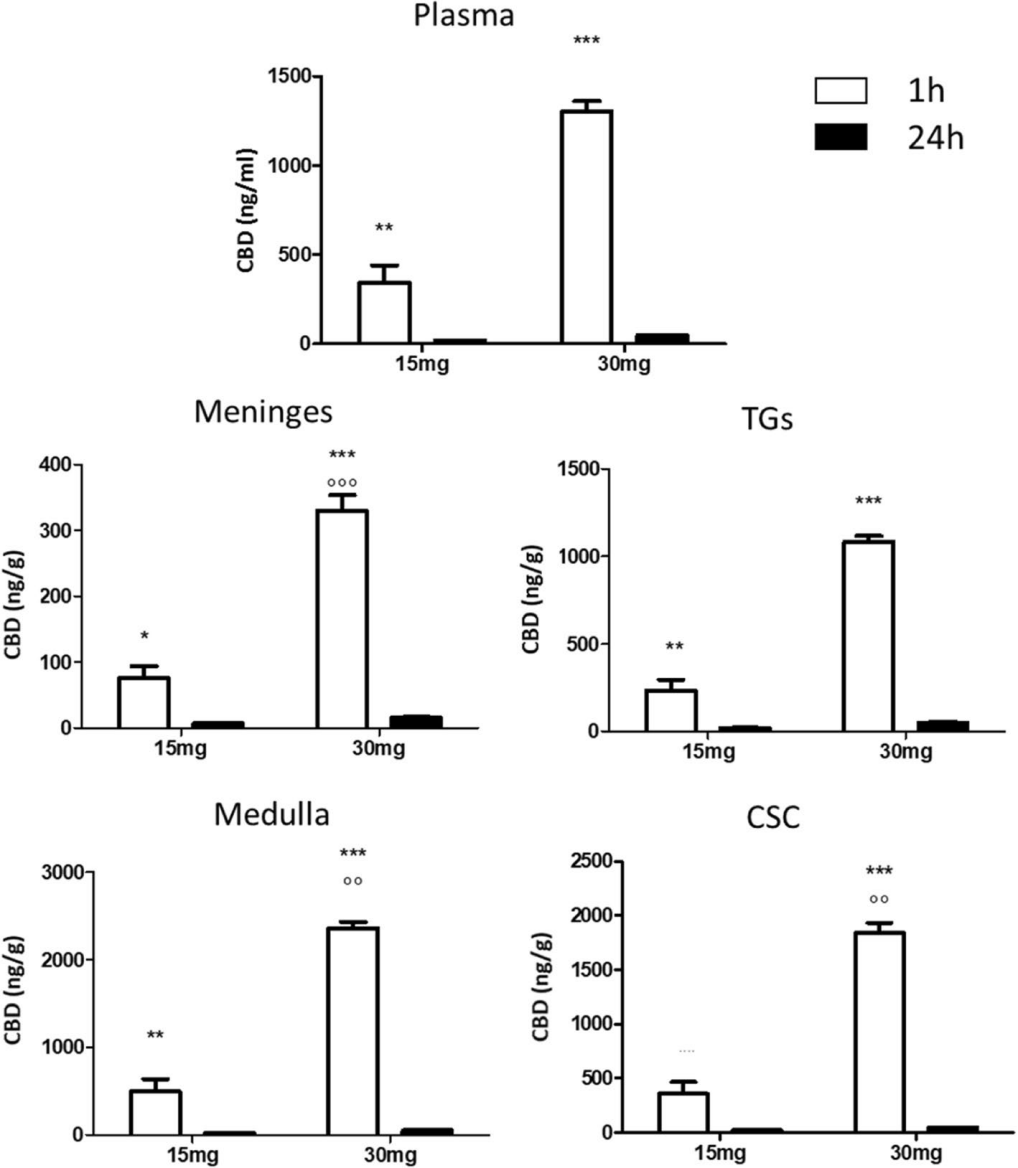
CBD levels in plasma, meninges, trigeminal ganglia (TGs), medulla and cervical spinal cord (CSC) in rats treated for 5 days with CBD 15 or 30 mg/kg, and sacrificed 1 h and 24 h after the last administration. Data were normally distributed and were analyzed by using 2-way ANOVA followed by Sidak's multiple comparisons test: *p < 0.05, **p < 0.01 and ***p < 0.001 vs. 24 h; °°*p* < 0.01 vs. 15 mg/kg. Data are expressed as mean ± SEM, *N* = 7 per group

One hour after the last CBD administration, CBD was detected in all areas under evaluation and in plasma, in a dose–response manner with a substantial decrease at 24 h post-treatment (Fig. 2), to demonstrate that CBD penetrates the brain but it does not accumulate in it.

## Acute migraine model

### Open field, grooming, rearing

**Fig. 3:**
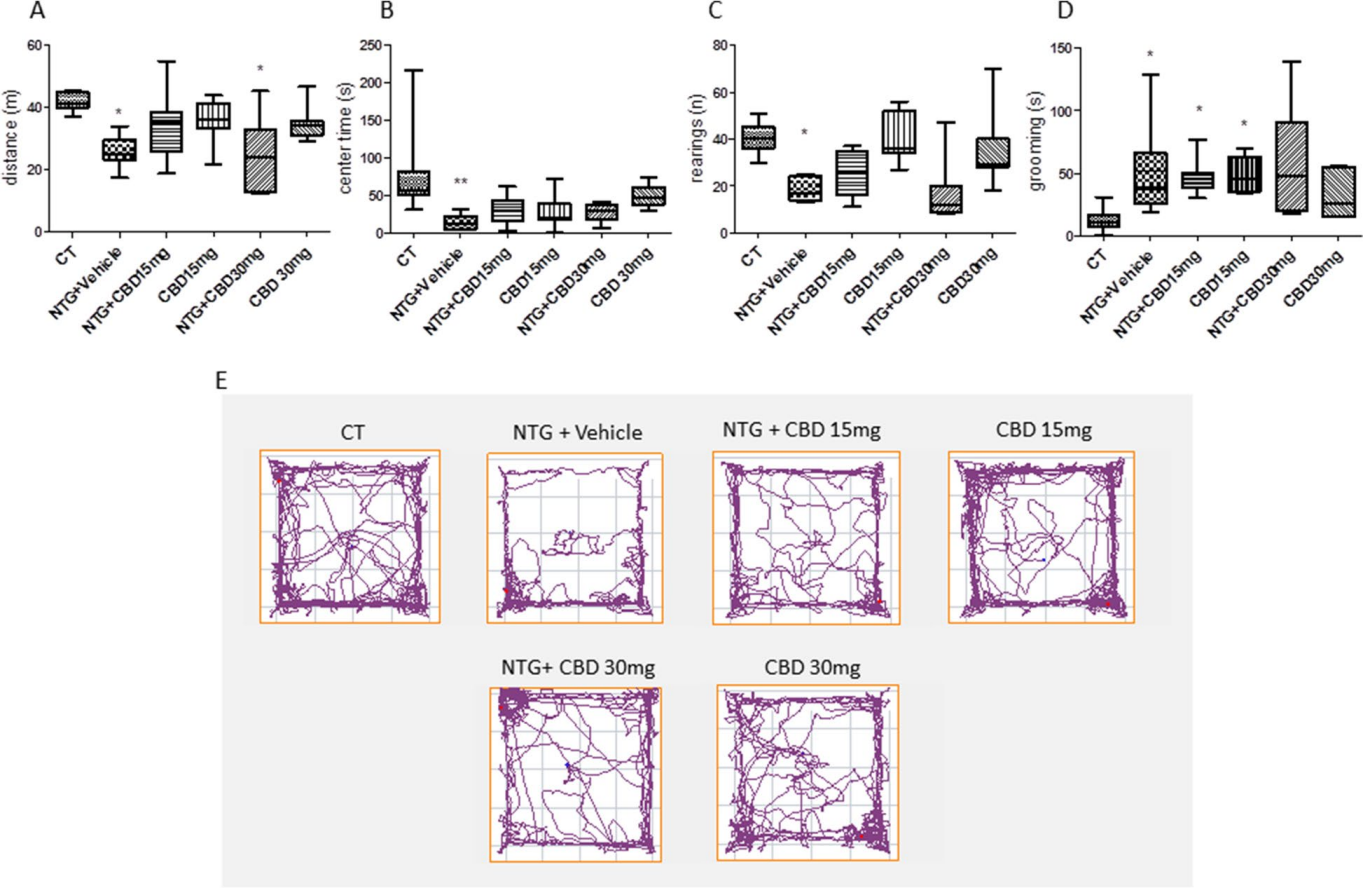
Open field test and grooming analysis in the acute migraine model. **A** Distance (expressed in meters) travelled in the apparatus; **B** time spent (expressed in seconds) in the center of the apparatus; **C** number of rearing; **D** time spent in grooming behavior (expressed in seconds); **E** Representative track plot reports recorded during the 10 min test sessions (ANY-maze). Data were not normally distributed and were analyzed using Kruskal–Wallis test, followed by Dunn’s post-hoc test: *p < 0.05 and **p < 0.01 vs. CT. Data were expressed as the median and the minimum and maximum values, *N* = 7–8 per group

Systemic administration of NTG reduced the locomotor activity, expressed as distance traveled compared with the control (CT) group (Fig. 3A). Additionally, it increased the anxiety-like behavior as indicated by a reduced time spent in the center of the open field and reduced the exploratory behavior, expressed as the number of rearing, compared with the CT group (Fig. 3B and C respectively). In the groups treated with CBD (15 or 30 mg/kg) prior to NTG, we observed a pattern toward attenuation of the NTG-induced behavioral modifications, which however did not reach statistical significance compared with the NTG group. No statistically significant changes were observed in the locomotor activity, time spent in the center of the open field, and exploratory behavior when CBD was administered alone (i.e. without the NTG challenge), although we detected a trend toward the reduction of center time with the lower CBD dose (Fig. 3B). As regards the grooming behavior, which is indicative of increased nociception, NTG significantly increased the time spent in grooming compared to the CT group and this effect was not affected by CBD administration (NTG + CBD group) (Fig. 3D). By contrast, CBD administered alone at the dose of 15 mg/kg significantly increased the time spent in grooming compared to the CT group (Fig. 3D). A similar pattern was also observed with the higher dose of CBD, without reaching a statistically significant level.

### Orofacial formalin test

**Fig. 4:**
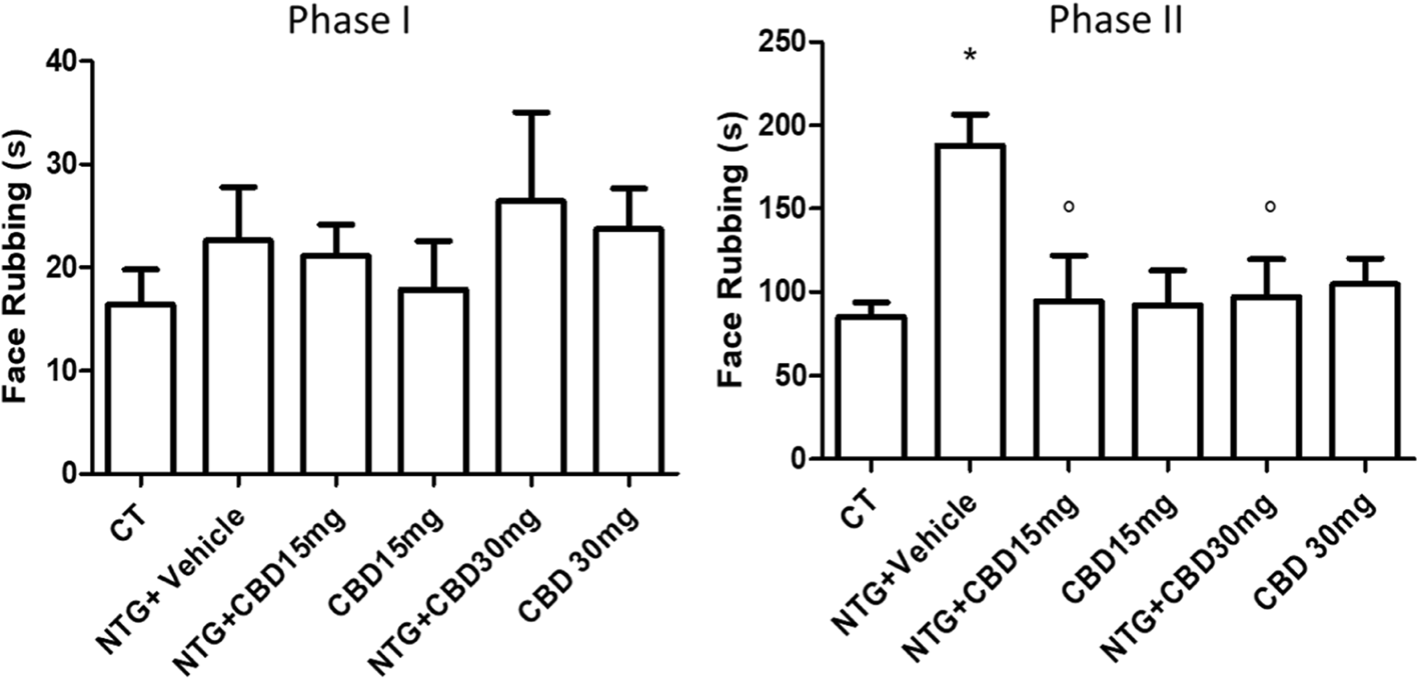
Orofacial formalin test in the acute migraine model; Data are expressed as mean time spent in face rubbing time (in seconds) in Phases I (0–3 min) and II (12–45 min). Data were normally distributed and were analyzed using one-way ANOVA followed by Tukey’s multiple comparisons test: *p < 0.05 vs CT; °*p* < 0.05 vs. NTG + Vehicle. Data are expressed as mean ± SEM, *N* = 7–8 per group

As illustrated in Fig. 4, NTG administration induced a hyperalgesic state, detectable as an increase in nocifensive behavior (total face rubbing time) during Phase II of the orofacial formalin test. CBD, at both doses, significantly reduced NTG-induced nocifensive behavior in Phase II. When CBD was administered with the NTG vehicle, no significant effect was detected compared with the CT group. No significant differences between groups were observed regarding Phase I of the test.

### Gene expression analysis

**Fig. 5:**
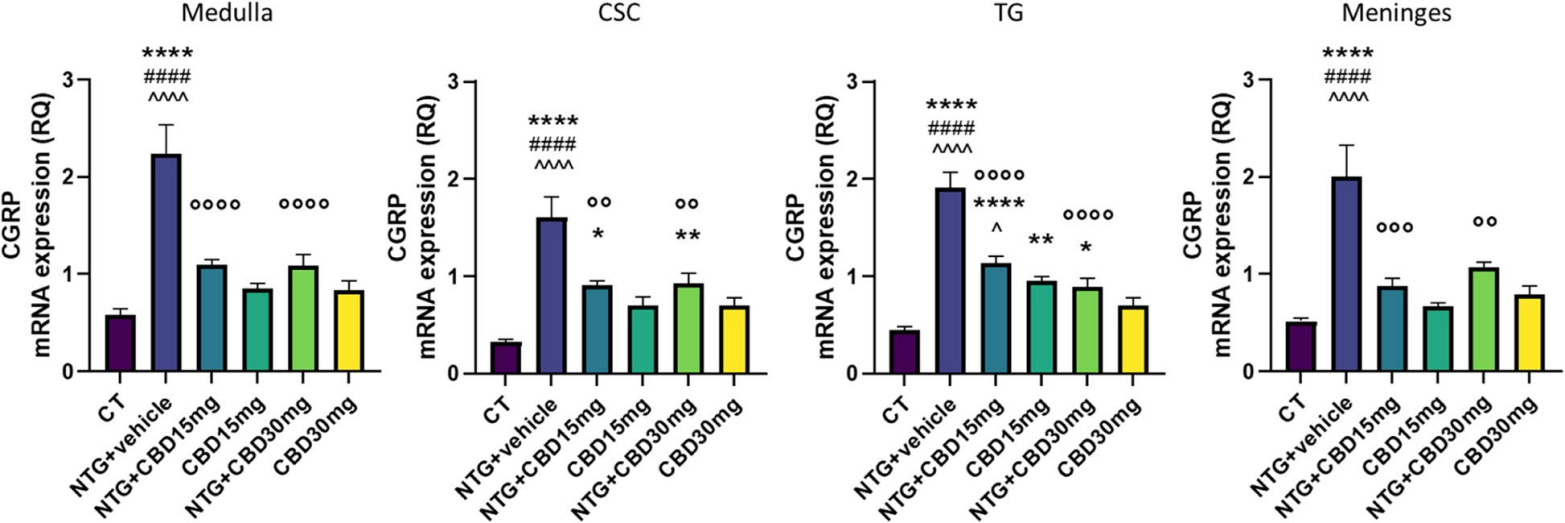
CGRP gene expression (expressed as relative quantification (RQ)) in medulla in toto, cervical spinal cord (CSC) and Trigeminal ganglion (TG) ipsilateral to formalin injection and in meninges, in the acute migraine model. Data were normally distributed and were analyzed using one-way ANOVA followed by Tukey’s multiple comparisons test:*p < 0.05, **p < 0.01 and ****p < 0.0001 vs. CT; °°*p* < 0.01, °°°*p* < 0.001 and °°°°*p* < 0.0001 vs. NTG; ####*p* < 0.0001 vs CBD 15 mg; ^*p* < 0.05 and ^^^^*p* < 0.0001 vs. CBD 30 mg. Data are expressed as mean ± SEM, *N* = 7–8 per group

**Fig. 6:**
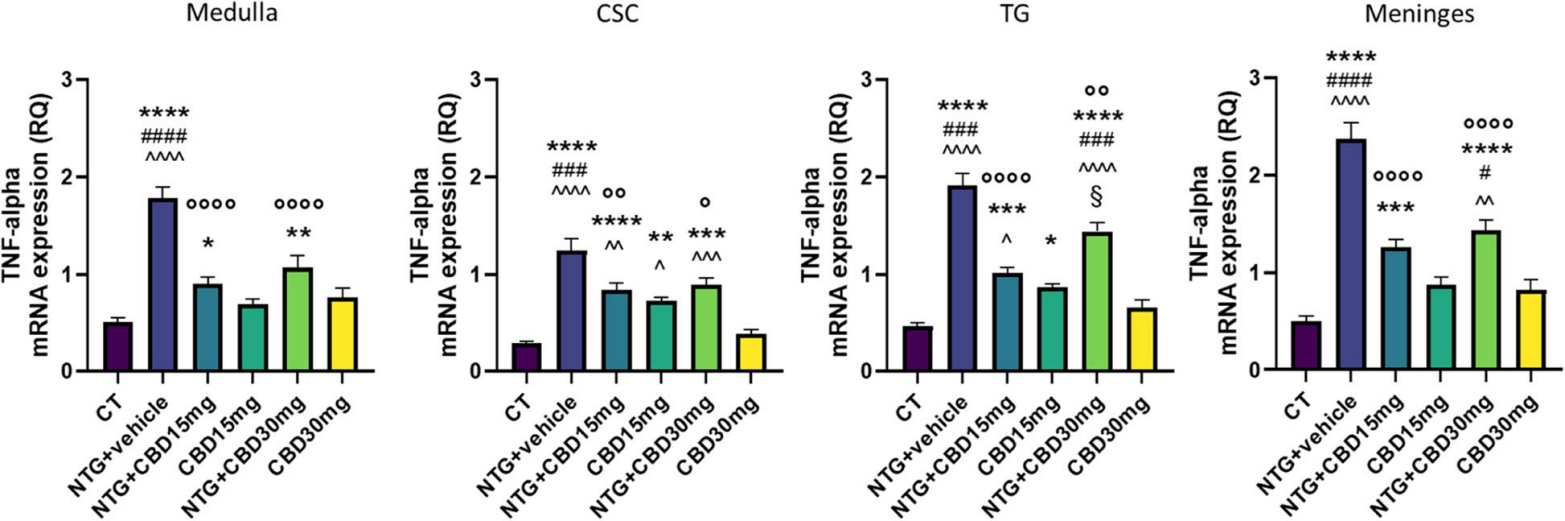
TNF-alpha gene expression (expressed as relative quantification (RQ)) in medulla in toto, cervical spinal cord (CSC) and Trigeminal ganglion (TG) ipsilateral to formalin injection and in meninges in the acute migraine model. Data were normally distributed and were analyzed using one-way ANOVA followed by Tukey's multiple comparisons test: *p < 0.05, **p < 0.01, ***p < 0.001 and ****p < 0.0001 vs. CT; °*p* < 0.05, °°*p* < 0.01 and °°°°*p* < 0.0001 vs. NTG; #*p* < 0.05, ###*p* < 0.001 and ####*p* < 0.0001 vs. CBD 15 mg; ^*p* < 0.05, ^^*p* < 0.01, ^^^*p* < 0.001 and ^^^^*p* < 0.0001 vs. CBD 30 mg; §*p* < 0.05 vs. NTG + CBD 15 mg. Data are expressed as mean ± SEM, *N* = 7–8 per group

**Fig. 7:**
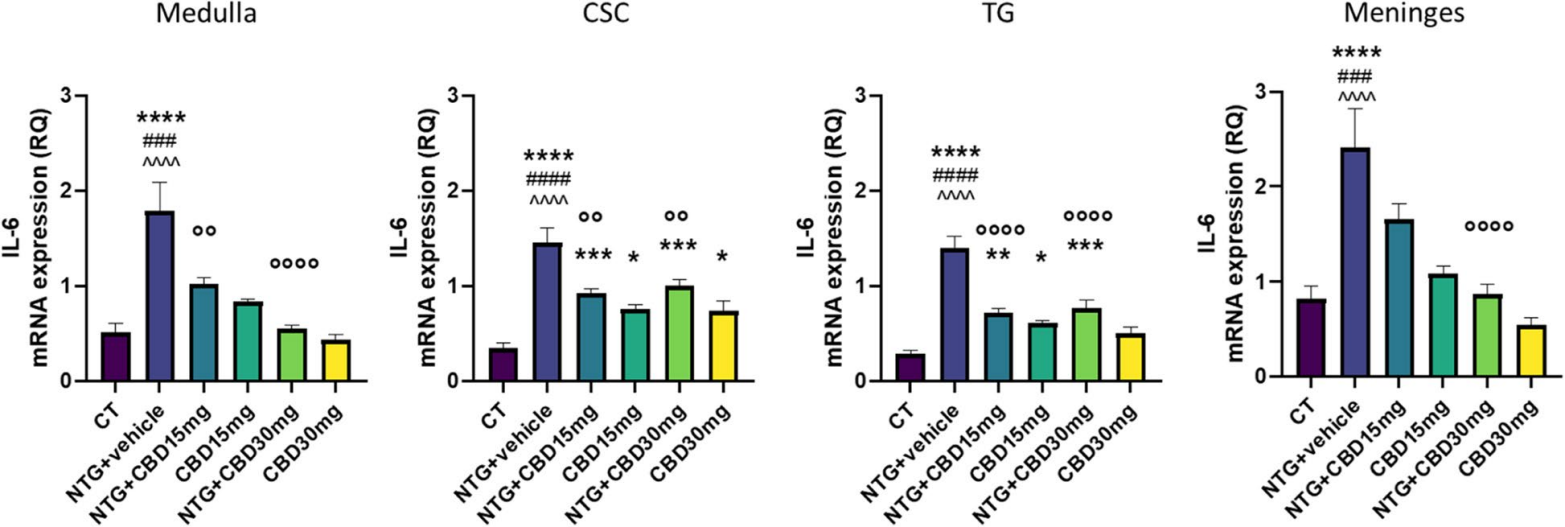
IL-6 gene expression (expressed as relative quantification (RQ)) in medulla in toto, cervical spinal cord (CSC) and Trigeminal ganglion (TG) ipsilateral to formalin injection and in meninges in the acute migraine model. Data were normally distributed and were analyzed using one-way ANOVA followed by Tukey's multiple comparisons test: *p < 0.05, **p < 0.01, ***p < 0.001 and ****p < 0.0001 vs. CT; °°*p* < 0.01 and °°°°*p* < 0.0001 vs. NTG; ###*p* < 0.001 and ####*p* < 0.0001 vs. CBD 15 mg; ^^^^ *p* < 0.0001 vs. CBD 30 mg. Data are expressed as mean ± SEM, *N* = 7–8 per group

**Fig. 8:**
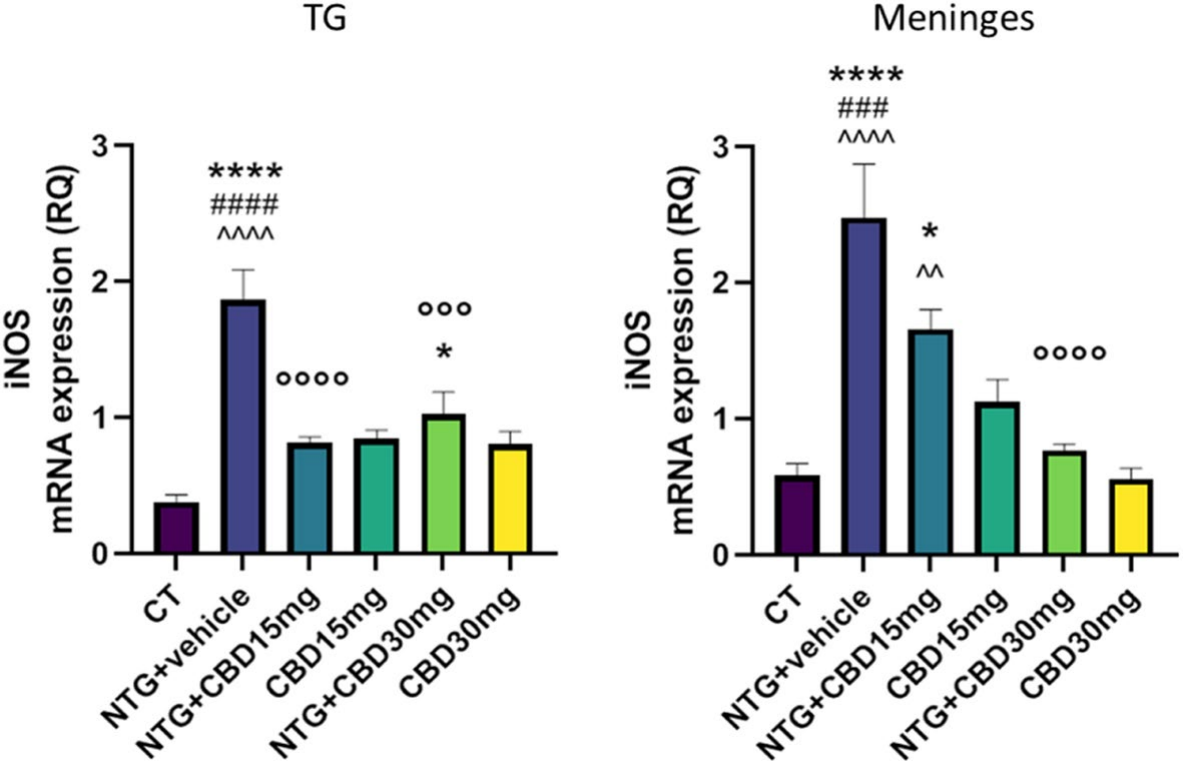
iNOS gene expression (expressed as relative quantification (RQ)) in TG ipsilateral to formalin injection and in meninges in the acute migraine model. Data were normally distributed and were analyzed using one-way ANOVA followed by Tukey's multiple comparisons test. *p < 0.05 and ****p < 0.0001 vs. CT; °°°*p* < 0.001 and °°°°*p* < 0.0001 vs. NTG; ###*p* < 0.001 and ####*p* < 0.0001 vs. CBD 15 mg; ^^*p* < 0.01 and ^^^^*p* < 0.0001 vs. CBD 30 mg. Data are expressed as mean ± SEM, *N* = 7–8-per group

NTG administration increased gene expression of CGRP (Fig. 5), Tumor necrosis factor-alpha (TNF-alpha) (Fig. 6), and interleukin-6 (IL)-6 (Fig. 7) in the medulla-pons, CSC and TG, and meninges when compared with the CT group. Additionally, NTG increased gene expression of iNOS in TG and meninges (Fig. 8). In the acute migraine model, CBD at both doses caused a significant decrease in pro-inflammatory cytokines, iNOS, and CGRP mRNA levels in all areas under evaluation (Figs. 5–8). By contrast, CBD when administered alone (without NTG) at the dose of 15 mg/kg induced a significant increase in cytokine gene expression in CSC and TG and an upregulation of CGRP in TG compared to the CT group. When CBD was administered alone at the higher dose (30 mg/kg) it did not induce any significant change.

### Chronic migraine model

#### Open field, grooming, rearing

**Fig. 9:**
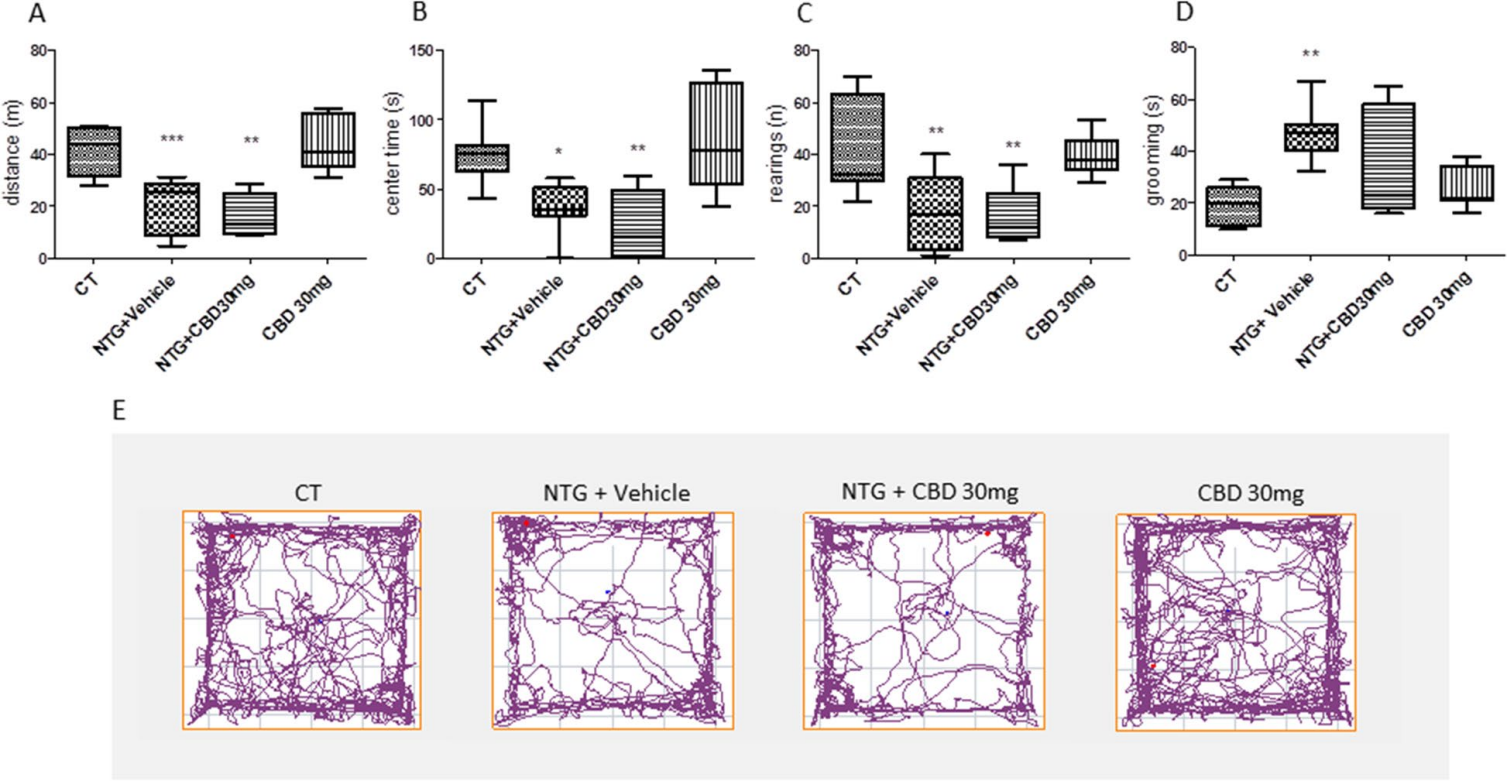
Open field test and grooming analysis in chronic conditions. **A** Distance (expressed in meters) travelled in the apparatus; **B** time spent (expressed in seconds) in the center of the apparatus; **C** number of rearings; **D** time spent in grooming behavior (expressed in seconds); **E** Representative track plot reports recorded during the 10 min test sessions (ANY-maze). Data were not normally distributed and were analyzed using Kruskal–Wallis test, followed by Dunn’s post-hoc test: **p < 0.01 and ***p < 0.001 vs. CT. Data were expressed as the median and the minimum and maximum values, *N* = 7 per group

Chronic administration of NTG decreased locomotor activity and exploration, expressed as the distance traveled and the number of rearing, compared with the CT group (Fig. 9A and C), similarly to the findings obtained in the acute migraine conditions. Additionally, NTG increased anxiety-like behaviors, as demonstrated by a decreased time spent in the open field's center (Fig. 9B and D). When CBD was associated with NTG treatment (NTG + CBD group), no effect was recorded on the locomotor activity, time spent in the center of the open field, and exploratory behavior compared to the NTG group.

Regarding grooming behavior, NTG significantly increased the time spent in grooming compared to the CT group, confirming a previous study [18], but also this activity was not affected by CBD administration (Fig. 9D). The group treated with CBD alone did not exhibit any discernible change from the CT group.

#### FAAH gene expression

**Fig. 10:**
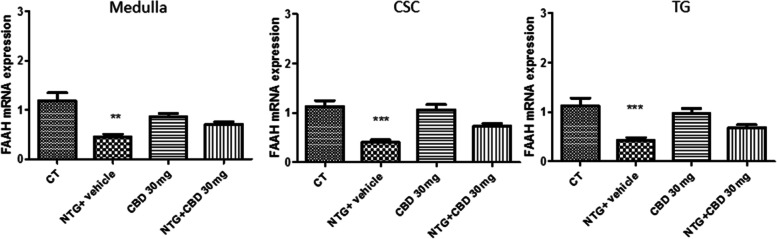
FAAH mRNA levels expressed as relative quantification (RQ) in medulla, cervical spinal cord (CSC) and trigeminal ganglion (TG) in chronic conditions. Data were normally distributed and were analyzed using one-way ANOVA followed by Tukey’s multiple comparisons test: ***p < 0.001 and **p < 0.01 vs. CT. Data are expressed as mean ± SEM, *N* = 7 per group

Chronic NTG treatment reduced FAAH gene expression in the central areas (medulla–pons, CSC) and in TG when compared with the CT group (Fig. 10). This decrease was not significantly reversed by chronic CBD treatment. No effect on FAAH gene expression was observed in rats injected with CBD alone (Fig. 10).

#### CGRP serum levels

**Fig. 11:**
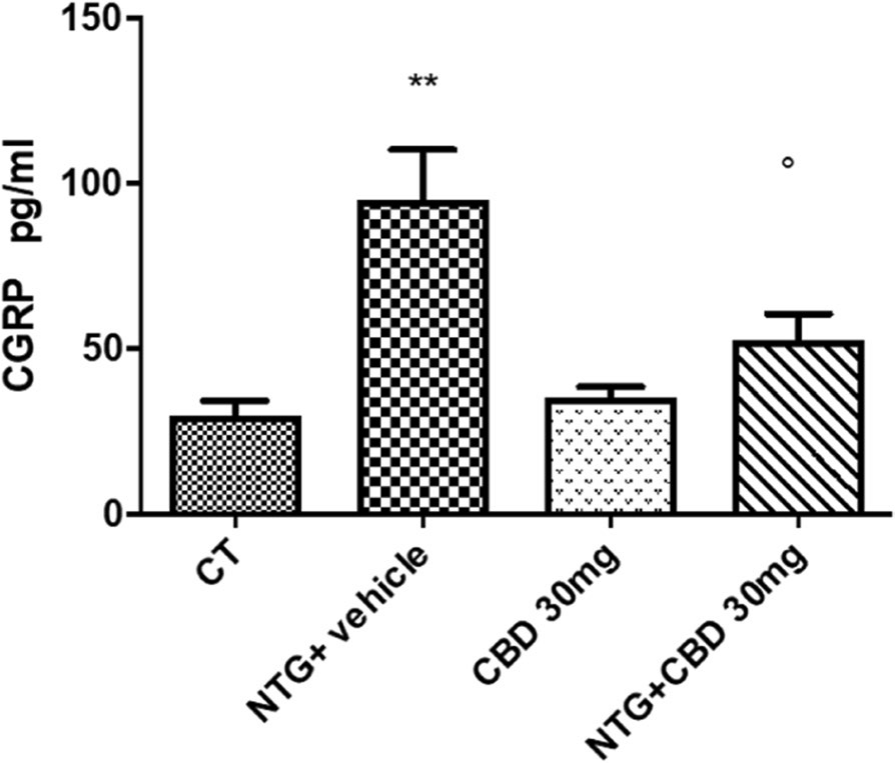
CGRP serum levels expressed as pg/mL in chronic conditions. Data were normally distributed and were analyzed using one-way ANOVA followed by Tukey’s multiple comparisons test: **p < 0.01 vs. CT and CBD 30 mg; °*p* < 0.05 vs. NTG. Data are expressed as mean ± SEM, *N* = 7 per group

Chronic NTG administration significantly increased CGRP serum levels compared with the CT group, confirming a previous study [34]. Chronic CBD treatment prevented this rise (Fig. 11). Serum levels of CGRP after chronic CBD were unaffected in the absence of NTG.

#### Cytokines tissue levels

**Fig. 12:**
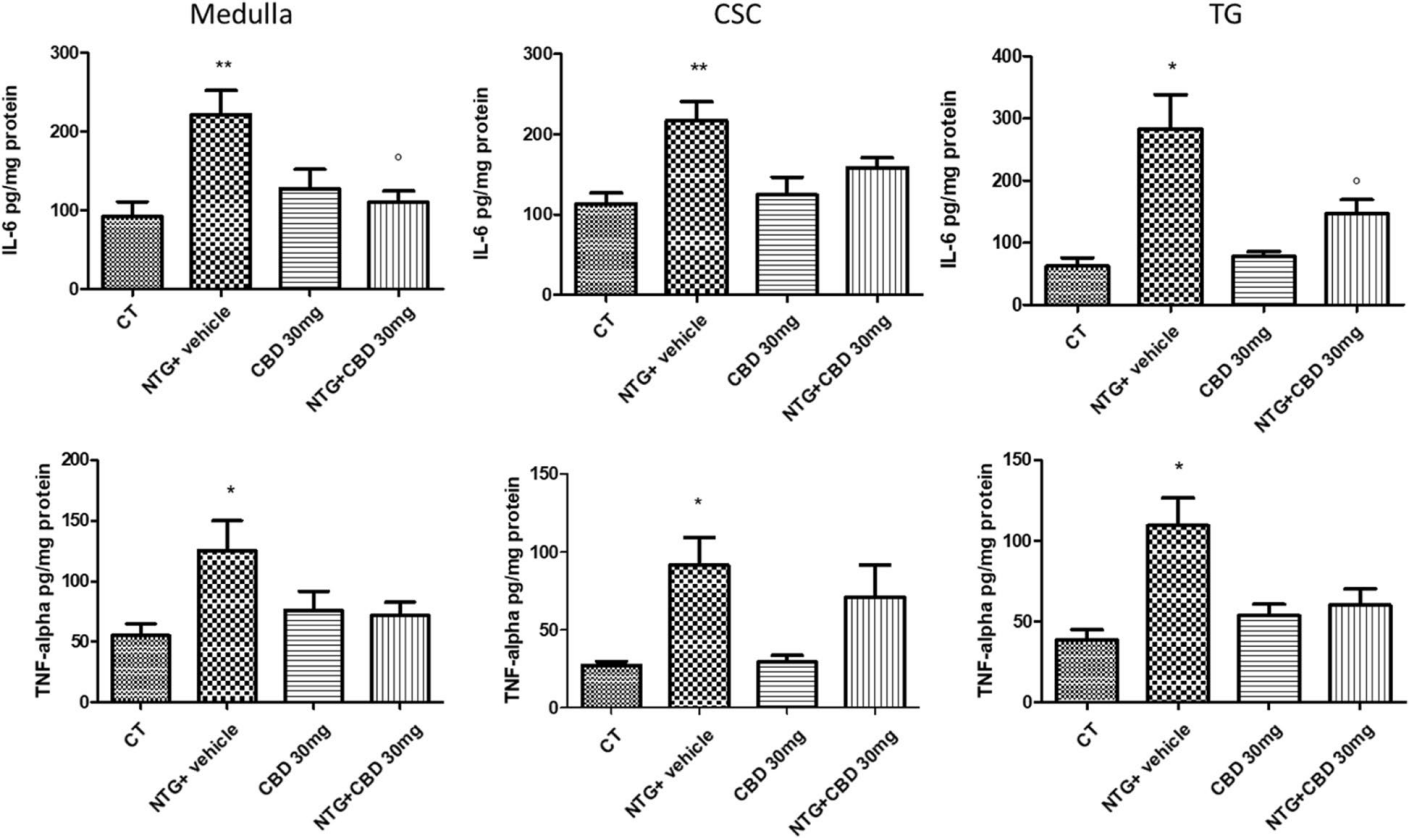
IL-6 protein and TNF-alpha protein levels (expressed as pg/mg of protein) in medulla, cervical spinal cord (CSC) and trigeminal ganglion (TG) in chronic conditions. Protein levels in tissues are expressed as pg/mg of protein. Data were normally distributed and were analyzed using one-way ANOVA followed by Tukey’s multiple comparisons test. *p < 0.05 and ** *p* < 0.01 vs. CT and CBD 30 mg; °*p* < 0.05 vs. NTG + Vehicle. Data are expressed as mean ± SEM, *N* = 7 per group

Chronic NTG treatment increased protein levels of TNF-alpha and IL-6 (Fig. 12) in medulla–pons, CSC, and TG compared to the CT group. CBD chronic treatment caused a significant decrease in NTG-induced protein IL-6 in the medulla–pons and TG, while no significant changes were observed in TNF-alpha protein levels in all the areas under investigation. CBD did not induce any significant change in the protein levels under evaluation when administered to the rats treated with NTG vehicle.

## Discussion

Preclinical and clinical evidence point to endocannabinoids and associated lipids, such as palmitoylethanolamide, having a role in migraine pathogenesis [35]. eCB tone modulation via inhibition of eCB-catabolizing enzymes has been a focus of research in animal models of migraine-related pain, suggesting that medications targeting the ES may be able to control migraine [12, 13]. CBD is one of the most important pharmacologically active phytocannabinoids and it is metabolically and chemically stable [36]. It is not psychotropic and has numerous pharmacological benefits, such as anti-inflammatory and antioxidant properties [20]. CBD has been considered a promising strategy against inflammation and neuropathic pain [24, 37]. It also belongs to a class of chemicals that have anxiolytic, depressive, antipsychotic, and anticonvulsant effects [5]. Cannabidiol's biological effects have been investigated, including the different molecular targets with which it interacts, such as cannabinoid receptors and other components of the ES [26, 38]. Like some other cannabinoids, CBD suppresses several mediators involved in migraine pain, such as cytokines and chemokines expression, reactive oxygen species and modulates immune cell system [28]. In a recent clinical trial, a mix of THC/CBD was tested in chronic migraine and chronic cluster headache patients suggesting positive effects [39]. Two clinical trials have begun but no findings have been published so far. The first investigates two doses of oral CBD on chronic migraine and the second evaluates the efficacy and safety of inhaled CBD on acute attacks [40]. To date preclinical and clinical studies that investigate the potential effect of CBD alone in migraine are lacking.

Here, we show beforehand that CBD reaches cranial areas of relevance for migraine and complement previous studies suggesting that CBD's apparent elimination half-life is between 4 to 24 h, and that after systemic treatment in rats, it is rapidly absorbed and distributed to the brain [41].

In the acute migraine model two doses of CBD (15 mg/kg and 30 mg/kg, i.p.) reduced NTG-induced trigeminal hyperalgesia together with a significant decrease in gene expression levels of iNOS, CGRP, and pro-inflammatory cytokines in migraine-relevant central and peripheral structures.

The findings suggest that CBD may be effective in the treatment of migraine pain, and are in agreement with previous data showing its potential efficacy in other types of pain. Indeed, CBD demonstrated potent anti-hyperalgesic effects on carrageenan-induced thermal hyperalgesia, reducing carrageenan-induced paw edema [42]. In this model, CBD also suppressed the overproduction of NO, endothelial NOS, and prostaglandins in paw tissues and cyclooxygenase (COX) activity [43]. Of note, hyperalgesia was significantly reduced by the low oral doses of CBD (5 and 7.5 mg/kg) and abolished by the higher doses (10, 20, and 40 mg/kg). By contrast, CBD (10 mg/kg, i.p.) treatment evoked antinociception only in phase I of the plantar formalin test in male mice but not in female, suggesting a differential effect [44]. The dose response observed in plasma, meninges, and brain concentrations of CBD was not reflected in the behavioral tests nor the expression of pro-inflammatory cytokines, iNOS, and CGRP mRNA. This non-linear dose effect for CBD in the neural/neurovascular areas involved in migraine pathophysiology possibly reflects a ceiling effect, already reached with the lower dose. This phenomenon is not new, since in a previous study evaluating the effect of CBD on stress-induced change on the hypothalamus–pituitary–adrenal axis, three different doses of CBD (5,15 and 30 mg/kg) intraperitoneally induced similar alterations in serotonin 5-HTR1A receptor gene expression in the amygdala and hippocampus [45]. Additionally, other studies conducted on different models/disease showed an unclear relationship between the dose of CBD and the biological effects [42, 46–48]. No significant effects were found on locomotor-exploratory-anxiety-like behavior and grooming alterations, induced by acute NTG administration after CBD treatment. Exploratory behaviors in baseline conditions were not altered by systemic CBD, indicating limited central effects of treatment. Of note, when CBD was used alone at dose of 15 mg/kg, we observed an increase in the time spent in grooming compared to the CT group, a change associated with a slight but significant increase of IL-6, TNF-alpha, and CGRP gene expression in the CSC and TG. In agreement, it was reported that the asocial BTBR mouse strain show an inflammatory profile, such as connections between inflammation and M1-related cytokines associated with repetitive grooming activity [49]. The findings are not in keeping with the analgesic effect of CBD but are not entirely surprising when considering previous data showing that CBD may increase IL-6 plasma levels, while decreasing the levels of IL-1β and IL-10. It suggests that CBD may modulate the inflammatory response [50] associated with grooming probably depending on the dose and the potential interaction with transient receptor potential vanilloid (TRPV) 1 [51, 52]. This latter is predominantly expressed in small trigeminal neurons, which play a key role in orofacial nociception [53].

In the chronic paradigm, we tested the higher CBD dose (30 mg/kg, i.p.) that did not show affect locomotor, exploratory, and anxiety-like behavior in rats injected with NTG or vehicle compared with the CT group. This finding seems in contrast with previous results where repeated administration of CBD at the same dose prevented the anxiogenic effect of 14 days of chronic unpredictable stress [54]. However, it is worth noting that other studies report no effect at low doses or an increase or a decrease in locomotor activity after high doses in mice [55, 56]. Whereas rats treated with CBD show hyperlocomotive effects after 10 and 30 mg/kg but only after 240–360 min post-administration [50, 57]. Thus, CBD's action on locomotor activity seems to depend on experimental settings and species, and probably reflects the activation of distinct pathways in the different settings, as if CBD was at the crossroads of multiple circuits.

CBD caused a significant decrease in NTG-induced IL-6 protein levels in the medulla–pons and TG. By contrast, it did not modulate TNF-alpha protein levels in the areas investigated. These findings suggest that CBD may modulate different inflammatory responses [50]. Several studies reported decreased TNF-alpha levels after CBD administration, while others showed unaltered TNF-alpha levels [24, 58, 59]. TNF-alpha levels were lowered in the frontal cortex following chronic CBD delivery, but did not change in the hippocampus after acute or chronic CBD administration [48]. In not neurological peripheral tissues, CBD decreases the pro-inflammatory profile of other cytokines [60]. Thus, once again, it seems that CBD effect on cytokine profile may be specific for the migraine animal model tested in this study.

Chronic NTG administration reduced FAAH gene expression in the central areas (medulla–pons, CSC) and TG compared with the CT group (NTG vehicle). This change has been interpreted as a dynamic compensatory mechanism for maintaining higher AEA levels after chronic NTG administration. In agreement, in a previous study, FAAH gene expression in the peripheral cells was significantly lower in migraine patients than in control subjects [61]. Chronic CBD administration did not change FAAH gene expression induced by NTG administration, suggesting that CBD activity in our experimental setting is not directly mediated by the inhibition of AEA catabolism, and that anti-migraine effects could be associated with other mechanisms [62]. CBD may inhibit AEA reuptake causing an increase in the concentration of available endogenous cannabinoids [26]. Moreover, it can activate TRPV receptors, directly or indirectly, by increasing the level of AEA, which is one of the endogenous TRPV1 agonists [38]. As results, AEA is able to increase CGRP release via TRPV1 activation and at the same time decrease CGRP release via Gi/o-coupled CB1 receptor activation or homologous and heterologous desensitization of TRPV1 receptors and transient receptor potential ankyrin 1, respectively [63, 64]. We did not measure AEA levels in this study, but we showed that CBD chronic treatment prevented the NTG-induced increase in CGRP serum levels. This activity is relevant since CGRP plays a significant role in migraine pathophysiology, because of its involvement in pain modulation and sensitization [34, 65]. Thus, we can speculate that CBD activity change on CGRP serum levels is related to the desensitization of TRPV1 receptors [66] although a previous in vitro study reported the lack of TRPV1 involvement in CBD-evoked CGRP release [52]. Indeed, other studies show that CBD is a TRPV2 ligand and can activate and subsequently desensitize this channel, confirming that CBD may have therapeutic potential against inflammatory and chronic pain [38]. Additionally, CBD may stimulate proliferator-activated receptor gamma (PPARγ) reducing inflammation [67]. PPARγ controls inflammation by triggering ubiquitination proteasomal degradation of p65, which inhibits the expression of pro-inflammatory genes such as COX-2 and some pro-inflammatory mediators such as TNF-alpha and IL-6, as well as nuclear factor-kappa B (NF-kB)-mediated inflammatory signaling [68]. As a result, CBD can reduce inflammation by blocking the NF-kB-mediated transcription of downstream genes [68].

### Strengths and limitations of the study

The present findings offer multifaceted pieces of information on the activity of CBD that is relevant for detecting and understanding its potential role in migraine pain. The methodology used is based on a solid and validated migraine-specific animal model [69, 70].

Some limitations are worth mentioning and call for additional research. Indeed, CBD is known to interact with multiple pathways at the peripheral and central levels. Here we did not evaluate the effect of CBD administration on endocannabinoid levels in the areas of interest and involvement of CB receptors, which would provide a more comprehensive view of the mechanisms or mediators involved in the CBD effect. Other mediators are likely involved in the complex biology underlying the activity of CBD in pain (e.g. TRPV receptors) which were not considered in this study. Additionally, we cannot exclude that the administration of CBD at different timings with respect to NTG might have a different impact on the outcome measures assessed in this study.

## Conclusions

In this study we provide documentation of the plasma and brain distribution of CBD and show that this phytocannabinoid—devoid of psychoactive activity—can modulate migraine-related nociceptive transmission and some inflammatory and pain mediators in migraine-specific animal models. Most preclinical studies show a significant analgesic effect of CBD, despite some methodological inconsistencies (i.e. different pain models, timings of treatment, route of administration). With these findings, we extend the potential field of application to migraine, setting the stage for future research and development studies that might lead to an additional treatment option for migraine.

## Data Availability

The data presented in this study are available on the ZENODO repository (https://doi.org/10.5281/zenodo.7780620).

## Abbreviations

CGRP: Calcitonin gene-related peptide
CBD: Cannabidiol
NTG: Nitroglycerin
CSC: Cervical spinal cord
CT: Control
COX: Cyclooxygenase
THC: Delta (9)-tetrahydrocannabinol
eCB: Endocannabinoid
ES: Endocannabinoid system
FAAH: Fatty acid amide hydrolase
AEA: Anandamide
GAPDH: Glyceraldehyde 3-phosphate dehydrogenase
SPE: Solid phase extraction
HPLC: High-performance liquid chromatography
MS/MS: Tandem mass spectrometry
iNOS: Inducible nitric oxide synthase
PPARγ: Peroxisome proliferator-activated receptor gamma
TRPV: Transient Receptor Potential Vanilloid
TG: Trigeminal ganglion
TGs: Trigeminal ganglia
NF-kB: Nuclear factor-kappa B
