# Correction: Characterization of the biochemical and behavioral effects of cannabidiol: implications for migraine

**DOI:** 10.1186/s10194-023-01605-1

**Published:** 2023-06-05

**Authors:** Rosaria Greco, Miriam Francavilla, Chiara Demartini, Anna Maria Zanaboni, Mikael H. Sodergren, Sara Facchetti, Barbara Pacchetti, Michela Palmisani, Valentina Franco, Cristina Tassorelli

**Affiliations:** 1 Unit of Translational Neurovascular Research, IRCCS Mondino Foundation, 27100 Pavia, Italy; 2 Department of Brain and Behavioral Sciences, University of Pavia, 27100 Pavia, Italy; 3 Curaleaf International, Guernsey, UK; 4 Medical Cannabis Research Group, Imperial College London, London, UK; 5 Clinical and Experimental Pharmacology Unit, Department of Internal Medicine and Therapeutics, University of Pavia, 27100 Pavia, Italy

**Correction: J Headache Pain 24, 48 (2023)**

**https://doi.org/10.1186/s10194-023-01589-y**

In this article [1], two errors were identified subsequent to the original publication.

The author name Michela Palmisani was incorrectly written as Michela Palmisan while the affiliation details for Michela Palmisani and Valentina Franco should read as follows:

Unit of Translational Neurovascular Research, IRCCS Mondino Foundation, 27100 Pavia, Italy,

Clinical and Experimental Pharmacology Unit, Department of Internal Medicine and Therapeutics, University of Pavia, 27100 Pavia, Italy.

The original article has been corrected.
